# PLAGL2 promotes bladder cancer progression via RACGAP1/RhoA GTPase/YAP1 signaling

**DOI:** 10.1038/s41419-023-05970-2

**Published:** 2023-07-15

**Authors:** Hualin Chen, Wenjie Yang, Yingjie Li, Zhigang Ji

**Affiliations:** Department of Urology, Peking Union Medical College Hospital, Chinese Academy of Medical Sciences and Peking Union Medical College, No.1 Shuaifuyuan Wangfujing Dongcheng District, Beijing, 100730 China

**Keywords:** Bladder cancer, Oncogenes

## Abstract

PLAGL2 is upregulated in various tumors, including bladder cancer (BCa). However, the mechanisms underlying the tumorigenic effects of PLAGL2 in BCa remain unclear. In our study, we proved that PLAGL2 was overexpressed in BCa tissues and correlated with decreased survival. Functionally, PLAGL2 deficiency significantly suppressed the proliferation and metastasis of BCa cells in vitro and in vivo. RNA sequencing, qRT‒PCR, immunoblotting, immunofluorescence staining, luciferase reporter, and ChIP assays revealed that overexpressed PLAGL2 disrupted the Hippo pathway and increased YAP1/TAZ activity by transactivating RACGAP1. Further investigations demonstrated that PLAGL2 activated YAP1/TAZ signaling via RACGAP1-mediated RhoA activation. Importantly, the RhoA inhibitor simvastatin or the YAP1/TAZ inhibitor verteporfin abrogated the proproliferative and prometastatic effects of BCa enhanced by PLAGL2. These findings suggest that PLAGL2 promotes BCa progression via RACGAP1/RhoA GTPase/YAP1 signaling. Hence, the core nodes of signaling may be promising therapeutic targets for BCa.

## Introduction

Bladder cancer (BCa) is one of the most common malignant tumors that develops in the urinary system, with approximately 570 thousand newly diagnosed cases and 200 thousand deaths worldwide in 2020 [1, 2]. Nearly 30% of BCa patients present with muscle-invasive tumors and advanced stage at initial diagnosis [3]. Furthermore, these patients have a decreased five-year overall survival rate of 60%, even when managed with standard of care (e.g., curative surgery) [4]. Therefore, new insights into the biological mechanisms of BCa progression will provide better therapeutic modalities and prolong survival.

Cancer is a multistep process, and many transcription factors (TFs) are required for carcinogenesis [5]. Dysregulated TFs are involved in various pro-oncogenic events, including proliferation, invasion, and metastasis, in various cancers, including hepatocellular carcinoma (HCC) [6], colorectal cancer (CRC) [7], glioma [8], and BCa [9]. Pleomorphic adenoma gene like-2 (PLAGL2), a member of the PLAGL gene family, is closely associated with the development of multiple cancers. For example, Yang et al. uncovered the pathological role of PLAGL2 in lung adenocarcinoma development [10]. The biological roles of PLAGL2 in promoting the progression of gastrointestinal cancer [11] and CRC [12] have also been addressed. One previous study by Qu et al. reported that PLAGL2 expression is highly correlated with prognosis and lymph node metastases of BCa [13]. However, the biological mechanisms of PLAGL2 in BCa progression remain unclear.

The Hippo pathway is evolutionarily conserved and regulates tissue growth and differentiation. Mounting evidence has indicated the crucial roles of the Hippo pathway in cancer cell proliferation, apoptosis, and metastasis [14, 15]. Within the core components of the Hippo pathway, upstream regulators such as MST1/2, SAV1, and LATS1/2 are tumor suppressors, whereas downstream effectors including YAP1/TAZ and TEADs are oncogenes [16]. The MST1/2 kinases with their binding partner SAV1 act to phosphorylate and activate LATS1/2 and its binding partner MOB1. The kinase module functions in a tumor suppressive manner and phosphorylates and inactivates the transcriptional module members of YAP1/TAZ, which are retained in the cytoplasm and subsequently degraded by the proteasome [17]. In many cancers, Hippo signaling is inhibited, and YAP1 and TAZ translocate into the nucleus to bind to the TEAD family. Activated TEADs regulate target genes such as CTGF and CYR61, which participate not only in tumor cell proliferation but also in metastasis [18]. Previous studies have uncovered the key roles of dysregulated TFs in the Hippo pathway. For example, enhanced FOXM1 promotes the radioresistance of cervical cancer cells by inhibiting Hippo signaling and increasing YAP1/TAZ activity [19]. In gastric cancer (GC), PAX6 knockdown eliminates resistance to CDK4/6 inhibitors by activating the Hippo signaling pathway [20]. However, the biological mechanisms of PLAGL2 in regulating the Hippo pathway are unknown.

In this study, we identified PLAGL2 as an oncogene that promotes BCa progression by inhibiting Hippo signaling. PLAGL2 is overexpressed in BCa tumor tissues and linked to decreased survival of patients with BCa. Functional studies demonstrated that upregulated PLAGL2 activates YAP1/TAZ and its target genes by disrupting Hippo signaling and thereafter contributes to enhanced BCa cell proliferation and metastasis both in vitro and in vivo. Mechanistically, we found that PLAGL2 transactivates its downstream target gene RACGAP1, a negative regulator of the Hippo pathway. RACGAP1 elicits ECT2-mediated RhoA GTPase activation, which leads to YAP1 activation. Thus, PLAGL2 facilitates BCa progression by regulating Hippo signaling, indicating its potential as a therapeutic target for BCa treatment.

## Materials and methods

### Clinical sample collection and immunohistochemistry staining (IHC)

The study protocol was approved by the Institutional Review Board of Peking Union Medical College Hospital. Informed written consent was obtained from each patient enrolled in this study. Fresh BCa tissues and pair-matched adjacent normal tissues were obtained from BCa patients who underwent radical cystectomy in our center and snap-frozen in liquid nitrogen immediately.

IHC was first incubated with primary antibodies against PLAGL2 (#11540-1-AP; Proteintech), RACGAP1 (#NBP1-33455; Novus), YAP1 (#12395; Cell Signaling Technology), and Ki-67 (#27309-1-AP; Proteintech) at 4 °C overnight. The sections were then incubated with secondary antibodies (Proteintech, Wuhan, China) at room temperature (RT) for 1 h and mounted in mounting medium with glycerol (Beyotime, Shanghai, China).

### Cell lines and cell culture

The BCa cell lines T24, 5637, J82, 253J and RT4 and the normal urothelial cell line SV-HUC-1 were purchased from the Cancer Institute of the Chinese Academy of Medical Sciences (Beijing, China). SV-HUC-1, T24, and J82 cells were cultured in DMEM, while 5637, RT4, and 253J cells were cultured in RPMI 1640. The plates were cultured at 37 °C in a humidified chamber with 5% CO_2_. All media and supplements were purchased from Beyotime Biotechnology. All basic media were supplemented with 10% FBS and 1% penicillin/streptomycin (Beyotime).

### Reagents

All primary and secondary antibodies used for immunoblotting were purchased from Proteintech. Primary antibodies included anti-PLAGL2, anti-GAPDH, anti-E-cadherin, anti-N-cadherin, anti-Slug, anti-Vimentin, anti-p-YAP1, anti-YAP1, anti-TAZ, anti-p-LATS1, anti-LATS1, anti-p-MST1, anti-MST1, anti-P84, anti-RACGAP1, anti-RhoA, and anti-ECT2. Simvastatin (a Rho GTPase inhibitor) and verteporfin (a YAP1 inhibitor) were obtained from Beijing Solarbio Science & Technology Co., Ltd. (Beijing, China).

### siRNA and plasmid transfection

Negative control siRNA (siNC) and gene-specific siRNAs (siPLAGL2, siRACGAP1, siLATS1, and siYAP1) were purchased from RiboBio (Guangzhou, China). The indicated cells were transfected with a mixture of siRNAs and Lipofectamine 3000 (Invitrogen, Carlsbad, CA, USA) according to the manufacturer’s protocols. The siRNA sequences are listed in Supplementary Table S1.

Empty vector (pcDNA3.1) and gene-specific plasmids (pcDNA3.1-PLAGL2/RACGAP1/LATS1) were obtained from RiboBio (Guangzhou, China). The pEZX-FR01-RACGAP1 promoter plasmid (RACGAP1-WT) and the corresponding mutant plasmids (RACGAP1-Muts 1-3) were purchased from GeneCopoeia, Inc. (Rockville, MD, USA). Sequencing was employed to determine the accuracy of plasmids before transfection.

### Quantitative RT‒PCR assay

Total RNA from cells was extracted with TRIzol (Life Technologies, Gaithersburg, MD, USA) following the manufacturer’s protocols. mRNAs were polyadenylated and reverse transcribed into cDNA, which was subsequently amplified and quantified. The target gene mRNA expression was normalized to GAPDH, the endogenous control. The primer sequences are listed in Supplementary Table S1.

### Western blotting analysis

In brief, the cells were lysed in RIPA lysis buffer with 1% protease and phosphatase inhibitors on ice. The whole cell lysates were centrifuged for 15 min at 12,000 rpm at 4 °C, and then, the supernatant was collected. Protein concentration was quantified by a BCA assay kit. SDS‒PAGE was used to separate the protein extracts, followed by PVDF membrane transfer. Then, PVDF membranes were immunoblotted with specific primary antibodies and secondary antibodies. All media and supplements were purchased from Beyotime Biotechnology.

### Chromatin immunoprecipitation (ChIP)

ChIP assays were performed using a ChIP Assay Kit purchased from Beyotime Biotechnology (Shanghai, China) following the manufacturer’s protocols. In brief, the indicated cells at a concentration of 2 × 10^6 cells/mL were treated with 1% formaldehyde at RT for 10 min to crosslink proteins to DNA. The cells were washed with ice-cold PBS containing protease inhibitors twice and then resuspended in SDS lysis buffer after centrifugation. The chromatin was sonicated for 6 min (30 s 12 times) after incubation at 4 °C for 15 min. The centrifuged supernatants were mixed with dilution buffer and incubated with anti-PLAGL2 antibody and protein G beads at 4 °C overnight. Coprecipitated DNAs were purified and quantified using PCR. The input was used to normalize all values. The primer sequences for the RACGAP1 promoter are listed in Supplementary Table S1.

### Dual-luciferase reporter assay

In brief, the dual-luciferase reporter assay was performed using the Dual-Luciferase® Reporter Assay System (Promega, Wisconsin, USA) according to the manufacturer’s instructions. The cells were seeded in a 24-well plate and cotransfected with 0.5 μg of RACGAP1-WT or RACGAP1-Mut promoter plasmid and 1.0 μg of pcDNA3.1-PLAGL2 or empty vector. The luciferase activity was measured after incubation for 48 h.

### RNA-seq

The detailed procedures have been reported in our previous study [3]. In brief, RNA sample preparation, library construction, and RNA sequencing were performed following the Illumina TruSeq RNA Sample Prep Kit protocol (Illumina, San Diego, CA, USA). Raw counts were mapped to the human genome GRCh38/hg38 by bowtie2.

### Nuclear and cytoplasmic extract preparation

The detailed procedures have been reported in a previous study [21]. In brief, cells were collected and lysed in 1 mL of Buffer A with cell pellet on ice for 10 min. The whole cell lysates were centrifuged for 3 min at 6500 rpm at 4 °C to pellet the nuclei. The cell pellet was lysed in IP buffer on ice for over 30 min, and the protein concentration was measured by a BCA kit.

### Cell proliferation and apoptosis assays

CCK-8 and colony formation assays were used to evaluate cell proliferation. For the CCK-8 assay, cells were seeded and cultured in 96-well plates for 48 h. The culture medium was replaced with CCK-8 solution, and the absorbance was measured at 450 nm after 4 h of incubation at 37 °C [22]. For the colony formation assay, cells were incubated in a 60-mm dish for two weeks and then fixed with paraformaldehyde for 20 min. After being washed with PBS, the colonies were stained with 0.5% crystal violet for 15 min. The cell proliferation was further evaluated by an EdU staining assay using an EdU assay kit (Beyotime) according to the following protocol. Briefly, in 12-well plates, 2 × 10^5 cells were seeded and incubated for 24 h. The cells were treated with 2% glycine and 0.5% Triton X-100 and stained with Apollo staining reaction buffer and DAPI. EdU-positive cells were analyzed by fluorescence microscopy.

Cell apoptosis was determined by flow cytometry assay as reported in previous studies [23]. In brief, cells were dissociated and resuspended at 1 × 10^6^ cells/mL in assay buffer and then cultured with 10 μL/ml JC-1 for 15 min at 37 °C. Cells were then analyzed by flow cytometry after washing twice with assay buffer.

### Cell invasion and migration assays

Wound-healing and transwell Matrigel invasion assays were employed to assess cell migration and invasion, respectively. Detailed procedures have been reported in our previous studies [3, 22]. Briefly, for the wound-healing assay, a wound was made with a 200 µL pipette tip, and the wound areas were photographed every 3 h to record cell migration. For the transwell Matrigel invasion assay, cells were seeded in the upper chamber, and 500 µL of complete medium was added to the lower chamber. Invasive cells under the filter were fixed, stained, and then counted.

### Lentivirus and shRNA transfection

Lentiviral vectors for human PLAGL2 gene overexpression (lenti-PLAGL2) and knockdown (lenti-shPLAGL2; 5'-GGAAATTTGTTCTAGATAA-3') were constructed by GeneChem (Shanghai, China). The corresponding empty vectors (lenti-Con and lenti-shCon) were used as negative controls. Then, 5637 (transfected by lenti-shPLAGL2 and lenti-shCon) and T24 cell lines (transfected by lenti-PLAGL2 and lenti-Con) were seeded in 24-well plates and transfected following the manufacturer’s protocols. The transfection efficacy was determined by qRT‒PCR and immunoblotting.

### Xenograft model and pulmonary metastasis model

All animal procedures were approved by the Institutional Animal Care and Use Ethics Committee of Peking Union Medical College Hospital. Four-week-old male BALB/c nude mice were purchased from WQJX BioTechnology (Wuhan, China). After a 7-day adaptation period, the nude mice were randomly divided into the indicated groups, with each group consisting of five mice.

For the xenograft model, stably transfected cells were suspended in 200 μL of PBS and subcutaneously injected into the flanks of nude mice. Every three days, tumor length (a) and width (b) were measured with a Vernier caliper, and tumor volumes were calculated using the formula ab^2^/2. Tumor nodules were harvested and weighed after the mice were sacrificed at the end of the fifth week.

For the pulmonary metastasis model, stably transfected cells were administered intravenously through the tail vein of nude mice. In the sixth week after cell injection, an IVIS Spectrum Imaging System (Caliper Life Sciences, Hopkinton, MA, USA) was utilized to detect pulmonary metastasis in mice and quantify the fluorescence intensity of lung metastases.

For evaluation of the impact of administering simvastatin and verteporfin on the proliferation and metastasis process in vivo, xenograft-bearing mice were randomized and subjected to treatment with either verteporfin (administered at a dose of 100 mg/kg body weight, via intraperitoneal injection, every 2 days) or simvastatin (administered at a dose of 5 mg/kg body weight, via intraperitoneal injection, 5 days per week) [24].

### Bioinformatics and data analysis

The distribution of PLAGL2 expression levels between normal and tumor tissues was analyzed in the UALCAN online resource (http://ualcan.path.uab.edu/index.html) [25]. Disease-free survival discrepancies between the high- and low-PLAGL2 groups were analyzed by the GEPIA online framework (http://gepia.cancer-pku.cn/index.html) [26]. Dysregulated pathways in siPLAGL2-transfected 5637 cell lines were decoded by the *enrichKEGG()* and *gseKEGG()* functions of the clusterProfiler R package [27]. The YAP signature was obtained from the Molecular Signatures Database (https://www.gsea-msigdb.org/gsea/msigdb/), and its activity score in the TCGA-BLCA cohort was determined by ssGSEA of the GSVA package [28]. The protein level of YAP-pS127 was downloaded from the GDC portal of the TCGA database. The Pearson correlations between PLAGL2 and RACGAP1, TP53BP2, CIT, and NPHP4 were analyzed in a curated GEO meta cohort, which was reported in our previous study [3].

### Statistical analysis

All values are presented as the means ± standard deviations (SD). Statistical differences between two groups were determined by unpaired Student’s *t* test via GraphPad 8.0.2 software (USA). Two-tailed *P* < 0.05 values were considered significant. All experiments were conducted three times.

## Results

### PLAGL2 is overexpressed in BCa

**Fig. 1:**
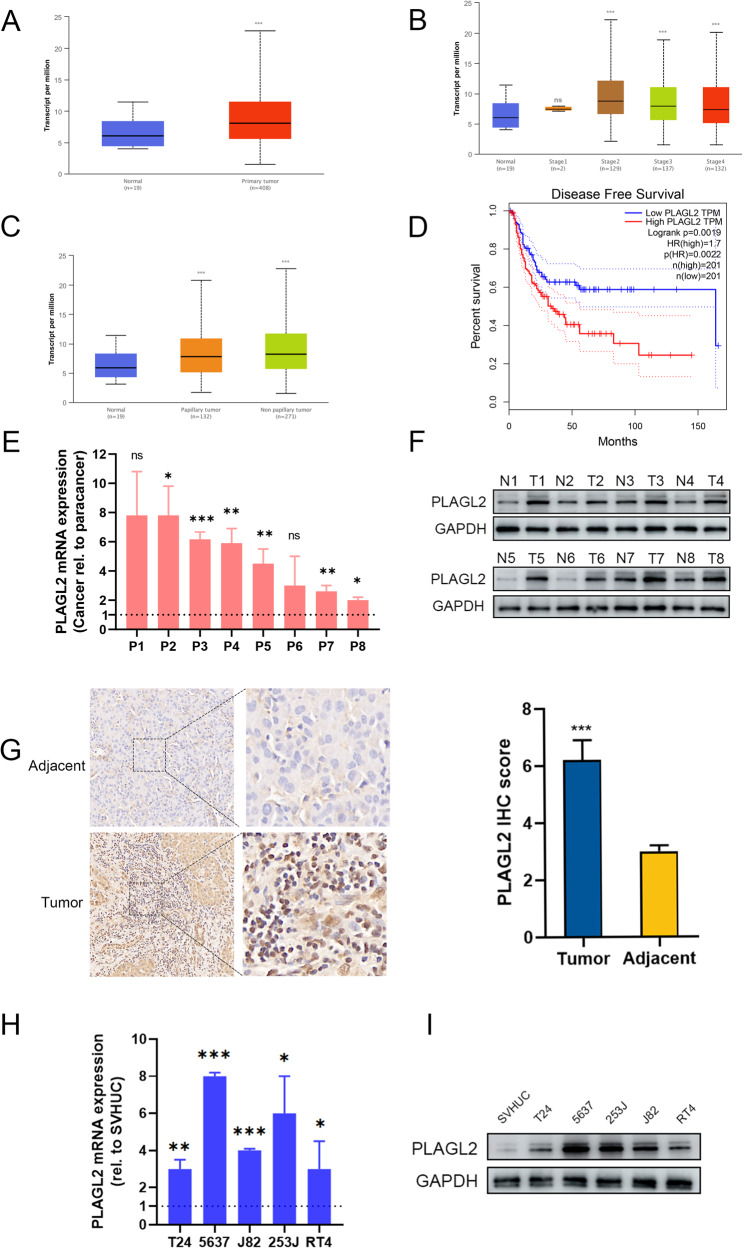
PLAGL2 is overexpressed in BCa. PLAGL2 mRNA levels in BCa and normal tissues (**A**), BCa tissues with different tumor stages (**B**), and different histological types (**C**). **D** Upregulated PLAGL2 was related to decreased DFS. PLAGL2 mRNA (**E**) and protein (**F**) levels in eight pairs of BCa and adjacent tissues. **G** Left panel: Representative IHC images of PLAGL2 in BCa and adjacent tissues. Right panel: Statistical quantification of the PLAGL2 IHC score. PLAGL2 mRNA (**H**) and protein (**I**) levels in the indicated cell lines. *P < 0.05, **P < 0.01 ***P < 0.001, ns no significance.

To explore the role of PLAGL2 in BCa, we analyzed the expression levels of PLAGL2 in the online UALCAN web resource, which contains 19 adjacent normal tissues and 408 primary tumor tissues [25]. The results showed that PLAGL2 was significantly upregulated in primary tumor tissues (Fig. 1A), regardless of tumor stage (except for stage I tumors vs. normal tissues, Fig. 1B) and histological type (Fig. 1C). In addition, overexpressed PLAGL2 was linked to decreased disease-free survival (*P* < 0.005, Fig. 1D).

To confirm this observation, we detected the PLAGL2 mRNA and protein levels in eight pairs of BCa tumor tissues and adjacent normal tissues. The results indicated elevated PLAGL2 in tumor tissues (Fig. 1E, F). In addition, PLAGL2 was highly expressed in the cellular nucleus of BCa tumor tissues, suggesting transcriptional regulation (Fig. 1G). Compared to those of the normal urothelial cell line SV-HUC-1, PLAGL2 mRNA levels were elevated in BCa cell lines, including T24, 5637, J82, 253J, and RT4 (Fig. 1H). Immunoblotting further showed relatively high PLAGL2 expression in 5637 and 253J cells and relatively low PLAGL2 expression in T24 and RT4 cells (Fig. 1I).

### PLAGL2 promotes the proliferation of BCa in vitro and in vivo

**Fig. 2:**
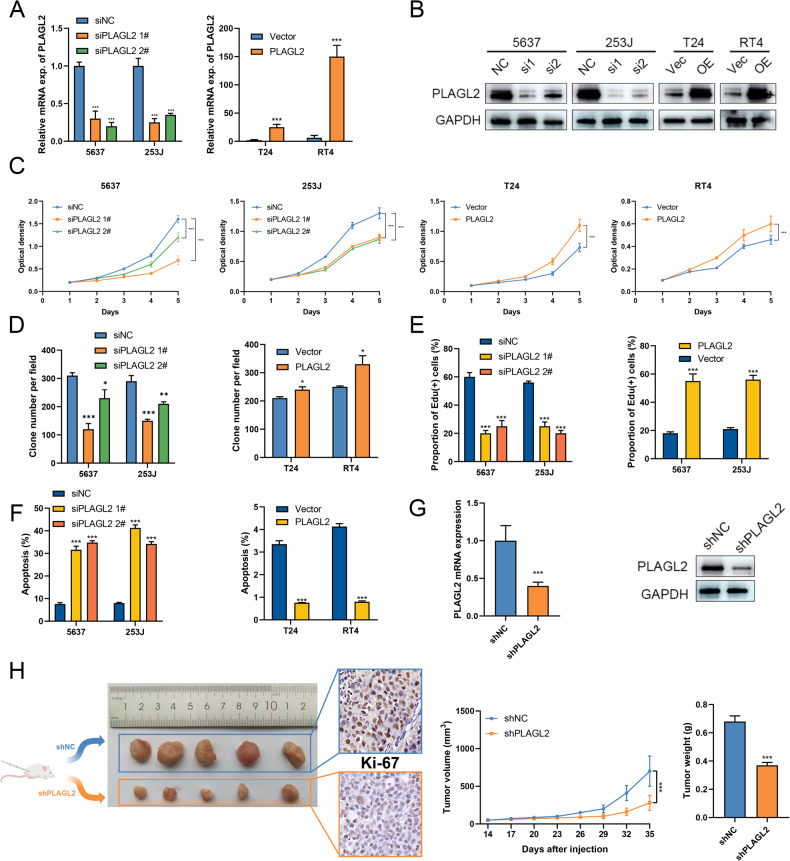
PLAGL2 promotes proliferation and inhibits apoptosis of BCa in vitro and in vivo. The efficiencies of siRNA and plasmid were verified by qRT‒PCR (**A**) and immunoblotting assays (**B**). CCK-8 (**C**), colony formation (**D**), and EdU assays (**E**) showed that knockdown of endogenous PLAGL2 expression in 5637 and 253J cells inhibited proliferation, whereas ectopic expression of PLAGL2 in T24 and RT4 cells promoted proliferation. **F** Flow cytometry assays showed the increased apoptosis rate by PLAGL2 overexpression and the reduced rate by PLAGL2 deficiency. **G** The efficiency of shRNA-constructed 5637 cell lines was verified by qRT‒PCR and immunoblotting assays. **H** Growth of xenografts in shNC- or shPLAGL2-transfected 5637 cells was measured every 3 days. Xenografts were dissected from nude mice at 35 days after injection with shNC or shPLAGL2-transfected 5637 cells, and the average weight of excised xenografts is shown. Ki-67 staining showed that shPLAGL2-transfected 5637 cells had decreased proliferation. *P < 0.05, **P < 0.01 ***P < 0.001.

To explore the biological role of PLAGL2 in BCa, we established BCa cell lines with PLAGL2 silencing (5637 and 253J) and overexpression (T24 and RT4) by siRNA and plasmid, respectively. qRT‒PCR and immunoblotting assays were used to validate the efficacy (Fig. 2A, B). CCK-8, colony formation, and EdU assays showed that PLAGL2 deficiency significantly inhibited, whereas PLAGL2 overexpression significantly promoted, the proliferation of BCa cells (Fig. 2C–E, Supplementary Fig. S1). To evaluate the effect of PLAGL2 on the apoptosis of BCa cells, flow cytometry showed that PLAGL2-deficient BCa cells had a significantly higher cell apoptosis rate than the negative control, whereas PLAGL2-overexpressing cells had a markedly reduced rate (Fig. 2F).

To further investigate the biological role of PLAGL2 in vivo, we established a 5637 cell line with stable PLAGL2 knockdown by LV-shPLALG2, and the efficacy was validated by qRT‒PCR and immunoblotting (Fig. 2G). Then, a xenograft assay was conducted by subcutaneously injecting the indicated 5637 cells into the left flank of nude mice under the same conditions. In the shPLAGL2 group, the tumor volume and weight were markedly reduced compared to those in the shNC group (Fig. 2H). In addition, the expression of Ki67 was significantly lower in the tumor tissues of the shPLAGL2 group.

### PLAGL2 promotes BCa metastasis in vitro and in vivo

**Fig. 3:**
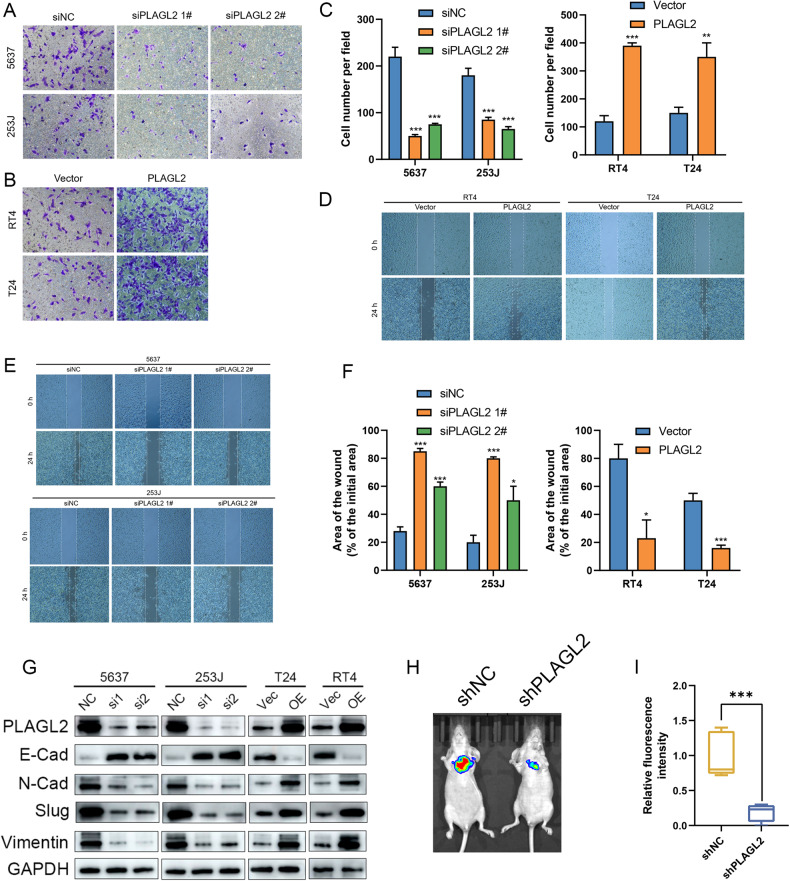
PLAGL2 promotes BCa metastasis in vitro and in vivo. Transwell invasion assays showed that knockdown of endogenous PLAGL2 expression in 5637 and 253J cells inhibited invasion (**A**, **C**), whereas ectopic expression of PLAGL2 in T24 and RT4 cells promoted invasion (**B**, **C**). Wound-healing assays showed that ectopic expression of PLAGL2 in T24 and RT4 cells promoted migration (**D**, **F**), whereas knockdown of endogenous PLAGL2 expression in 5637 and 253J cells inhibited the migration of cells (**E**, **F**). **G** EMT-related gene expression was assessed by Western blotting in the indicated cells. **H** Nude mice were injected with 5637-shPLAGL2/5637-shNC cells six weeks after injection, and lung fluorescence imaging was performed to detect lung metastases. **I** Fluorescence intensity quantification in lung metastasis. *P < 0.05, **P < 0.01 ***P < 0.001.

An in vitro transwell Matrigel assay was conducted to evaluate the effect of PLALG2 interference on the invasion of BCa cells. The results showed that siPLAGL2 significantly inhibited the invasion of 5637 and 253J cells (Fig. 3A, C), whereas PLALG2 overexpression promoted the invasion of T24 and RT4 cells (Fig. 3B, C). Similar trends were found in the wound-healing assay. The migration rate was increased in PLAGL2-overexpressing T24 and RT4 cells (Fig. 3D, F) and decreased in PLAGL2-deficient 5637 and 253J cells (Fig. 3E, F).

Epithelial to mesenchymal transition (EMT) is a key process in the carcinogenesis of BCa, contributing to tumor invasiveness and distant metastasis [29]. Next, we investigated the effect of PLAGL2 on EMT by immunoblotting. PLAGL2 knockdown was related to upregulation of the epithelial marker E-cadherin and downregulation of mesenchymal markers (N-cadherin, Slug, and Vimentin), whereas PLAGL2 overexpression exhibited the opposite effect (Fig. 3G).

To evaluate the effect of PALGL2 on cell metastasis in vivo, we established pulmonary metastatic models by injecting LV-shPLAGL2 and LV-shNC stable 5637 cells into the tail vein of nude mice. The results showed that knockdown of PLAGL2 expression suppressed lung metastasis in vivo (Fig. 3H, I). These findings confirm that PLAGL2 is critical for BCa cell metastasis.

### PLAGL2 activates YAP1 by inhibiting Hippo signaling

**Fig. 4:**
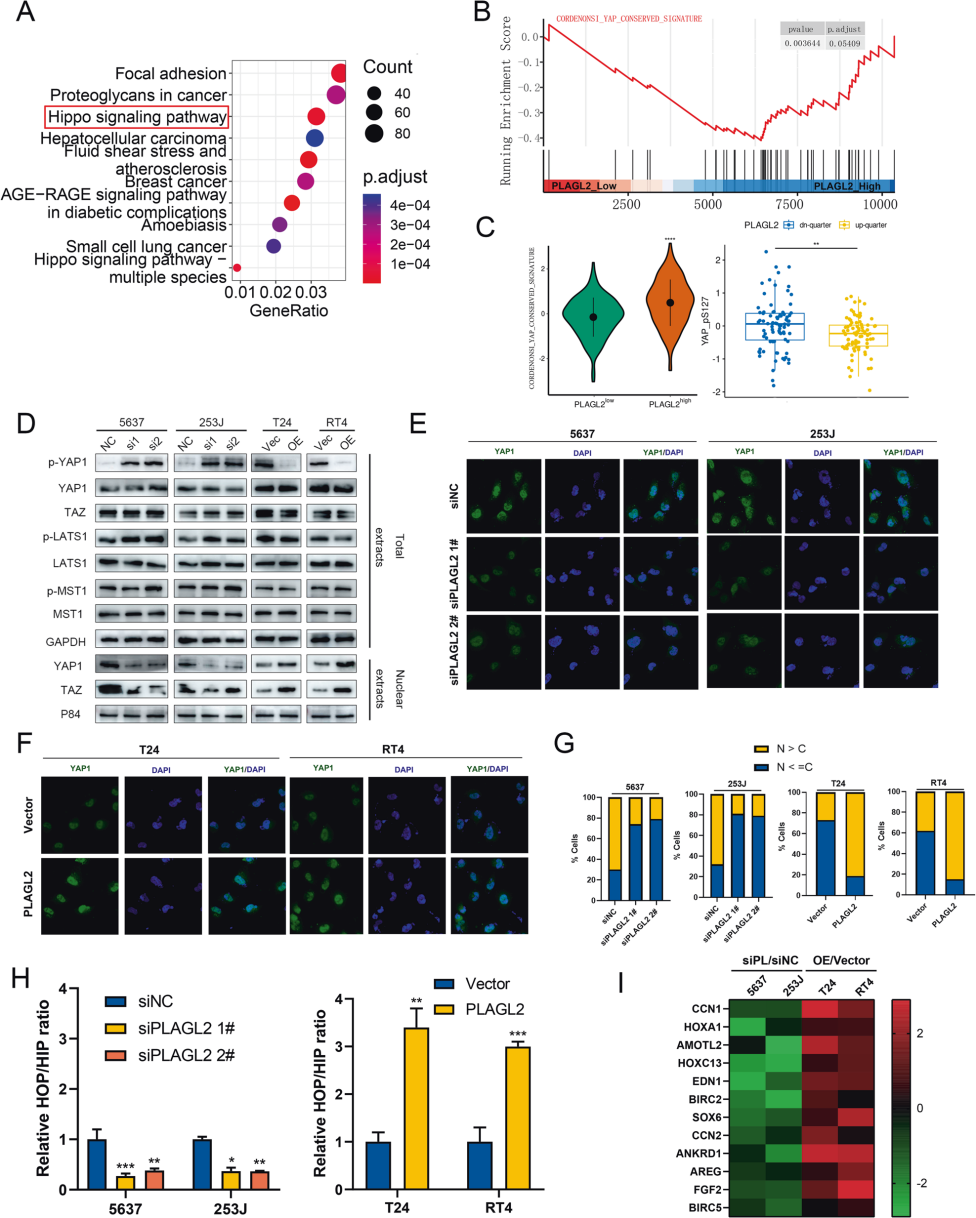
PLAGL2 activates YAP1 by inhibiting Hippo signaling. KEGG enrichment analysis (**A**) and GSEA (**B**) of the RNA-seq data in 5637-siPLAGL2 versus 5637-siNC cells. YAP1 signaling was enriched and increased in PLAGL2-overexpressing cells. **C** Left panel: Distribution of enrichment scores of the YAP signature between high- and low-PLAGL2 expression groups (TCGA-BLCA, transcriptome profiling). Right panel: Distribution of YAP-pS127 expression between the highest and lowest quartiles of PLAGL2 (TCGA-BLCA, proteome profiling). **D** Immunoblotting assays of the indicated protein expression in the indicated cells after PLAGL2 interference. GAPDH and P84 were used as loading controls for total and nuclear extracts, respectively. **E**–**G** Immunofluorescence assays indicated the expression and subcellular localization of YAP1 in the indicated cells after PLAGL2 interference. The percentage of subcellular localization of YAP1 in the indicated cells is shown. N nucleus, C cytoplasm. **H** HOP/HIP luciferase reporter assays showed YAP1/TAZ-TEAD transcriptional activity in the indicated cells after PLAGL2 interference. **I** The fold change in mRNA levels of the indicated genes was calculated by log2 transformation in qRT‒PCR analysis, comparing cells overexpressing PLAGL2 (OE) versus cells with vector or siPLAGL2 (siPL) versus siNC. *P < 0.05, **P < 0.01, ***P < 0.001, ****P < 0.001.

To investigate the mechanisms underlying PLAGL2-induced BCa progression, we subjected total RNA from the PLAGL2 knockdown 5637 cell line and control cells to RNA sequencing. The top ten enriched terms by the differentially expressed genes (provided in Table S2) are presented in Fig. 4A. The Hippo/YAP1 signaling pathway was enriched. A similar finding was observed through GSEA, as shown in Fig. 4B. By analyzing TCGA-BLCA transcriptome data, we found that the enrichment activity score of the YAP signature determined by ssGSEA was also increased in the high PLAGL2 expression group (Fig. 4C). In addition, the protein levels of p-YAP1-S127 (phosphorylated YAP1, inactivate YAP1) were decreased in the high PLAGL2 expression group (Fig. 4C). These results indicated that overexpressed PLAGL2 could activate YAP1 activity.

To confirm this, we performed immunoblotting to demonstrate that PLAGL2 inhibition increased the levels of p-YAP1 and p-LATS1 and decreased the nuclear levels of YAP1 and TAZ, whereas PLAGL2 overexpression had the opposite effect, without affecting the levels of p-MST1 (Fig. 4D). In addition, PLAGL2 inhibition inhibited, whereas PLAGL2 overexpression enhanced, the nuclear translocation of YAP1, as illustrated by fluorescence immunostaining (Fig. 4E–G).

In many cancers, the active kinase cascades of the Hippo pathway are inhibited, and YAP1/TAZ translocates into the cellular nucleus and binds to TEADs to transcriptionally activate target genes. Therefore, to further evaluate the effect of PLAGL2 on the transcriptional activity of YAP1/TAZ-TEAD, we performed HOP/HIP flash luciferase assays and revealed that the luciferase activities were significantly reduced by PLAGL2 inhibition but increased by PLAGL2 overexpression (Fig. 4H). As expected, the typical downstream target genes of YAP1/TAZ, including ANKRD1, CCN2 (CTGF), and CCN1 (Cyr61), were decreased in PLAGL2 knockdown cells but were upregulated significantly after ectopic expression of PLAGL2 (Fig. 4I).

To further investigate the mechanisms underlying PLALG2 regulation of the Hippo pathway, we performed a series of rescue assays. Notably, LATS1 deletion restored the YAP1/TAZ-TEAD reporter activity and expression of downstream targets (ANKRD1, CCN1, and CCN2) suppressed by PLAGL2 deficiency (Supplementary Fig. S2A, B). However, enhanced LATS1 expression inhibited the luciferase activity and downstream expression elevated by PLAGL2 overexpression (Supplementary Fig. S2A, B). Similar findings were observed in rescue CCK-8 (Supplementary Fig. S2C), colony formation (Supplementary Fig. S2D), EdU (Supplementary Fig. S2E), apoptosis (Supplementary Fig. S2F), transwell invasion (Supplementary Fig. S2G), and wound-healing assays (Supplementary Fig. S2H). Together, these findings revealed that PLAGL2 activates YAP1/TAZ activity by inhibiting the Hippo pathway.

### PLAGL2 transcriptionally activates RACGAP1 to repress Hippo signaling

Previous studies have reported several negative regulators of the Hippo pathway, including RACGAP1, TP53BP2, CIT, and NPHP4 [30–33]. We have proven that PLAGL2 increases YAP1/TAZ activity by inhibiting p-LATS1 (not p-MST1) in the Hippo pathway. Since PLAGL2 functions as a transcription factor that localizes to the nucleus and transcriptionally regulates downstream targets, we further investigated whether PLAGL2 could modulate these negative regulators to disrupt Hippo signaling.

**Fig. 5:**
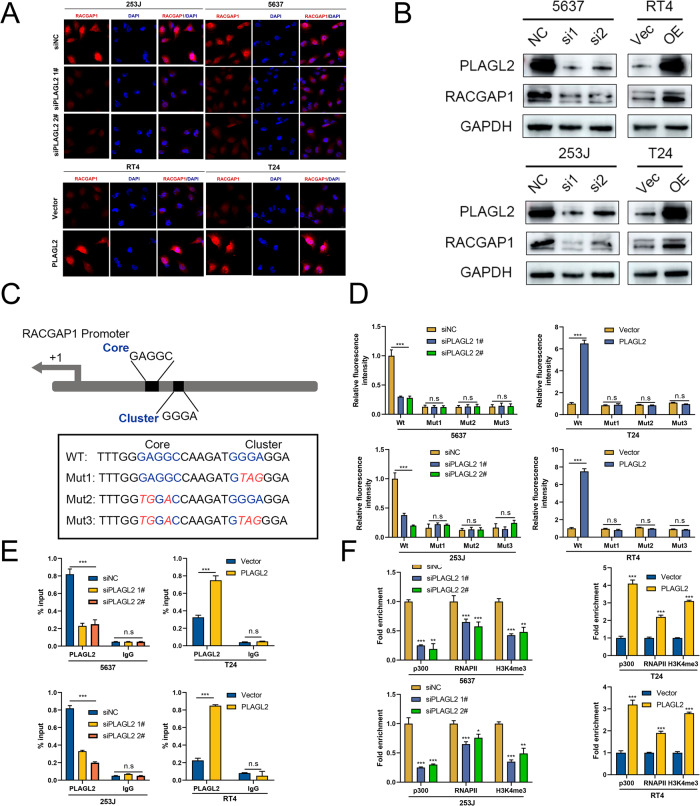
PLAGL2 transcriptionally activates RACGAP1 to repress Hippo signaling. Immunofluorescence (**A**) and immunoblotting assays (**B**) indicated the expression of RACGAP1 in the indicated cells after PLAGL2 interference. **C** Schematic representation of the RACGAP1 promoter. The sequences of wild-type/mutant PLAGL2 binding sites are indicated. **D** Luciferase activity assays were performed in the indicated cells transfected with wild-type (RACGAP1-WT) or mutant-type (RACGAP1-mut) RACGAP1 promoter-reporter plasmids. **E** ChIP‒qPCR analysis displays RACGAP1 enrichment on the promoter of RACGAP1. IgG indicates the negative control. **F** ChIP assays were employed in the indicated cells using anti-p300, anti-RNA polymerase II, and anti-H3K4me3 antibodies. *P < 0.05, **P < 0.01, ***P < 0.001, n.s no significance.

Intriguingly, a strong positive correlation between PLAGL2 and RACGAP1 was observed by analyzing the GEO meta cohort, which was curated in our previous study [3] (Supplementary Fig. S3A). qRT‒PCR verified that RACGAP1 mRNA levels were elevated in PLAGL2-overexpressing BCa cells but decreased in PLAGL2-deficient BCa cells (Supplementary Fig. S3B). Similar trends were observed in immunofluorescence staining and Western blotting assays (Fig. 5A, B). Therefore, we hypothesized the underlying regulatory mechanism between PLAGL2 and RACGAP1, considering the close correlations. Given the transcriptional role of PLALG2, it might bind to the promoter of RACGAP1 to modulate its expression levels and further regulate the Hippo pathway. Previous studies have revealed that the consensus binding sequence of PLAGL2 is GRGGC(N)6-8RGGK [34]. Interestingly, we identified one potential PLAGL2 transcriptional binding site in the RACGAP1 promoter region (Fig. 5C). A luciferase activity assay was employed to determine the transcriptional regulation of RACGAP1 by PLAGL2 by transfecting promoter-reporter plasmids that harbored wild-type and mutant binding sites (Muts 1-3) into BCa cells with the indicated PLAGL2 expression levels. The results revealed that PLAGL2 overexpression significantly increased the luciferase activity of the wild-type RACGAP1 promoter but had no effect on mutant promoters (Fig. 5D). In addition, both the core and the G-cluster were essential to the transcriptional regulation of RACGAP1 by PLAGL2. The ChIP assay also revealed that PLAGL2 was bound to the RACGAP1 promoter region (Fig. 5E). Furthermore, we investigated three molecules—transcriptional coactivator p300 acetyltransferase, RNA polymerase II, and the transcription-activating mark H3K4me3—and observed that inhibited PLAGL2 reduced, whereas enhanced PLAGL2 increased, the enrichment of these molecules on the RACGAP1 promoter (Fig. 5F).

**Fig. 6:**
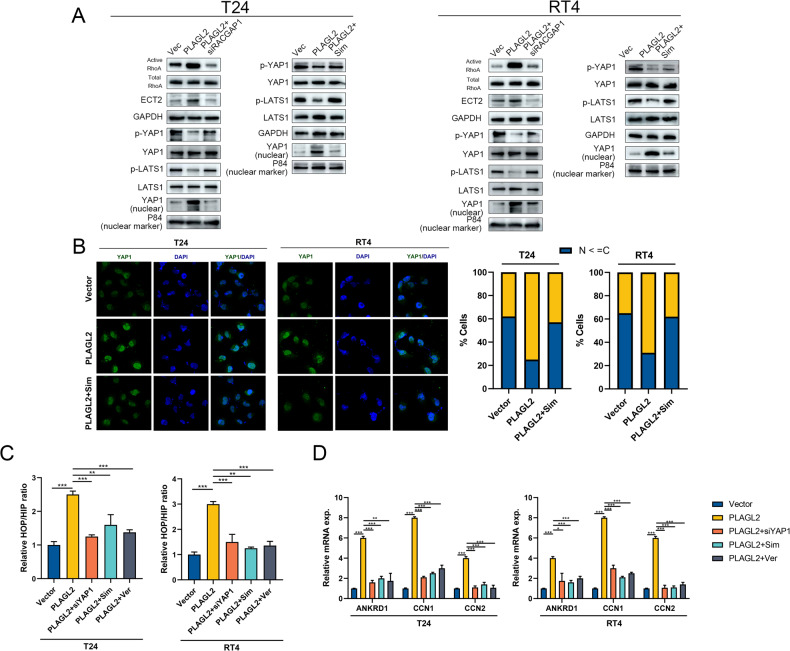
PLAGL2 promotes YAP1 signaling by activating RhoA. **A** Immunoblotting assays of the indicated protein expression in T24 and RT4 cells after PLAGL2, RACGAP1, and simvastatin (5 μM, 24 h) interference. GAPDH and P84 were used as loading controls for total and nuclear extracts, respectively. **B** Immunofluorescence assays indicated the expression and subcellular localization of YAP1 in T24 and RT4 cells after PLAGL2 interference and simvastatin treatment. The percentage of subcellular localization of YAP1 is shown. N nucleus, C cytoplasm. **C** HOP/HIP luciferase reporter assays showed YAP1/TAZ-TEAD transcriptional activity in T24 and RT4 cells after PLAGL2 interference and simvastatin/verteporfin treatment. **D** The mRNA levels of YAP1-targeted genes in T24 and RT4 cells after PLAGL2 interference and simvastatin/verteporfin treatment. *P < 0.05, **P < 0.01, ***P < 0.001.

Subsequently, a series of rescue assays were employed to demonstrate the effect of RACGAGP1-mediated PLAGL2 inhibition of the Hippo pathway. Significantly, RACGAP1 knockdown increased the protein levels of p-YAP1 and p-LATS1 but decreased the increase in nuclear YAP1 induced by PLAGL2 overexpression (Fig. 6A). Similarly, RACGAP1 knockdown abrogated the increase in YAP1/TAZ-TEAD promoter activity and downstream target gene mRNA levels induced by PLAGL2 overexpression (Supplementary Fig. S3C, D). Conversely, PLAGL2 deficiency-induced decreased promoter activity and target gene expression were restored by RACGAP1 overexpression (Supplementary Fig. S3C, D). Consistently, silencing RACGAP1 significantly reduced the proliferation, invasion, and migration of PLAGL2-overexpressing BCa cells, while overexpressing RACGAP1 significantly restored these abilities induced by PLAGL2 knockdown (Supplementary Fig. S3E–J). These results reveal that PLAGL2 activates YAP1/TAZ signaling by promoting RACGAP1 transcription, contributing to BCa progression.

### PLAGL2 promotes YAP1 signaling by activating RhoA

Yang et al. revealed that RACGAP1 could increase the activity of ECT2-mediated RhoA GTPase (active RhoA), promote the formation of filamentous actin (F-actin), reduce activation of the Hippo pathway, and activate YAP1/TAZ signaling [33]. Moreover, Varzav and colleagues elucidated the mechanisms by which RhoA promotes YAP1/TAZ activation by inhibiting the LATS kinase cascade [35]. Therefore, we hypothesized that PLAGL2 might inhibit LATS1 activity and activate YAP1/TAZ signaling via the RACGAP1/ECT2/RhoA GTPase/LATS1 axis.

Strikingly, RACGAP1 inhibition abrogated the protein expression of ECT2 and active RhoA elevated by PLAGL2 overexpression (Fig. 6A). Next, we observed that simvastatin, an inhibitor of RhoA GTPase, restored the protein levels of p-YAP1 and p-LATS1 (which indicated the activated Hippo pathway) inhibited by PLAGL2 overexpression (Fig. 6A). In addition, the nuclear YAP1 protein levels were decreased in PLAGL2-overexpressing BCa cells treated with simvastatin (Fig. 6A, B). Furthermore, YAP1/TAZ-TEAD luciferase assays showed that simvastatin could reverse the reporter activity promoted by PLAGL2 expression, similar to the effects of YAP1 deficiency by siRNA or the inhibitor verteporfin (Fig. 6C). Similar trends were found in the expression levels of YAP1/TAZ downstream targets (Fig. 6D). Together, our findings revealed that PLAGL2 increases YAP1/TAZ activity through the RACGAP1/ECT2/RhoA GTPase/LATS1 axis.

### Simvastatin and verteporfin abrogate PLAGL2-mediated BCa progression

**Fig. 7:**
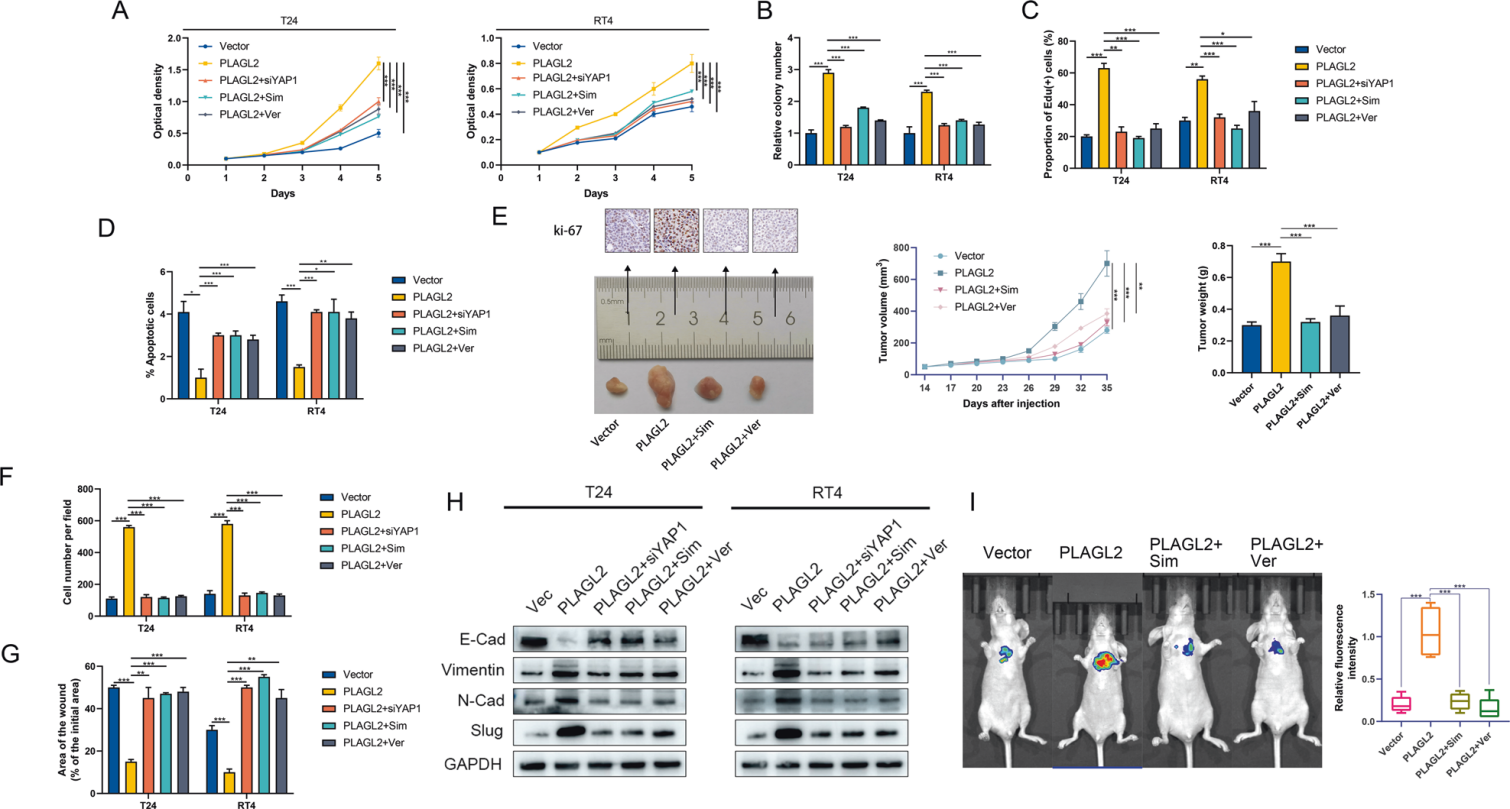
Simvastatin and verteporfin abrogate PLAGL2-mediated BCa progression. Simvastatin and verteporfin reversed the proproliferative effects of BCa cells induced by PLAGL2 overexpression as indicated by CCK-8 (**A**), colony formation (**B**), and EdU assays (**C**). **D** Simvastatin and verteporfin increased the apoptosis rate of BCa cells reduced by PLAGL2 overexpression. **E** Growth curves of xenografts in T24 cells after PLAGL2, simvastatin, and verteporfin interference. Xenografts were dissected from nude mice 35 days after cell injection, and the average weight of excised xenografts is shown. Ki-67 staining showed the proliferative capacity of T24 cells in each group. Simvastatin and verteporfin reversed the prometastic effects of BCa cells induced by PLAGL2 overexpression, as indicated by transwell invasion (**F**) and wound-healing assays (**G**). **H** EMT-related gene expression was assessed by Western blotting in the indicated cells. **I** Left panel: Lung fluorescence imaging was performed to detect lung metastases. Right panel: fluorescence intensity quantification in lung metastasis. *P < 0.05, **P < 0.01, ***P < 0.001.

To further evaluate the roles of RACGAP1-mediated RhoA activation and YAP1 signaling in the biological phenotype of BCa cells regulated by PLAGL2, we employed a series of in vitro and in vivo phenotypic assays. In the in vitro experiments, either RhoA GTPase inhibition by simvastatin or YAP1 deficiency remarkably reversed the proliferative and antiapoptotic effects of PLAGL2 on BCa cells (Fig. 7A–D). In vivo experiments further revealed that simvastatin or verteporfin treatment dramatically reversed the tumor volume and weight increase induced by PLAGL2 overexpression (Fig. 7E).

Similar trends were observed in transwell invasion and wound-healing migration assays (Fig. 7F, G). In addition, as presented by Western blotting, the protein expression of the epithelial marker E-cadherin was restored, whereas the mesenchymal marker protein levels were reversed by simvastatin or verteporfin (Fig. 7H). An in vivo lung metastasis mouse model further confirmed that administration of simvastatin or verteporfin inhibited lung metastasis in tumor-bearing mice (Fig. 7I). These results collectively demonstrated that RACGAP1-mediated RhoA activation and YAP1 signaling are indispensable for MYBL2-induced progressive effects on BCa cells.

### The PLAGL2/RACGAP1/YAP1 axis in BCa: clinical relevance

**Fig. 8:**
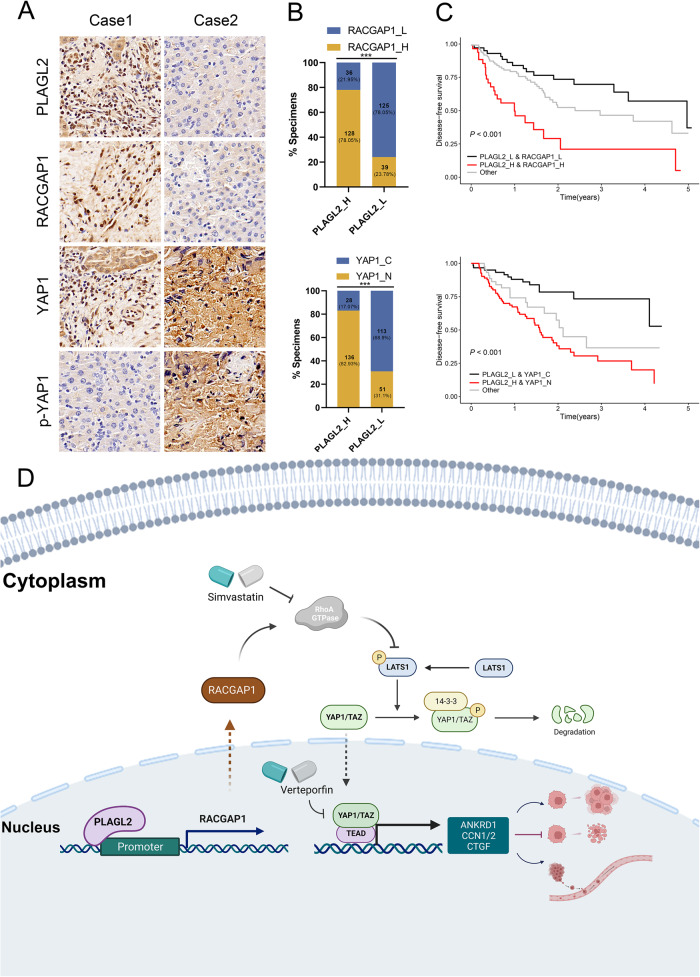
Clinical relevance of the PLAGL2/RACGAP1/YAP1 axis in human BCa. **A** Representative IHC images of PLAGL2, RACGAP1, YAP1, and p-YAP1 in 164 BCa specimens. **B** Upper panel: The proportion of specimens with the indicated RACGAP1 between those with high and low PLAGL2 expression. Bottom panel: The proportion of specimens with the indicated nuclear YAP1 between those with high and low PLAGL2 expression. **C** DFS of BCa patients with the indicated expression of proteins. **D** The schematic diagram illustrates that PLAGL2 promotes BCa progression via RACGAP1-mediated RhoA activation. ***P < 0.001.

To determine the clinical correlation of PLAGL2 with YAP1 signaling activity in clinical BCa tissues, we examined the protein levels of RACGAP1, p-YAP1, and nuclear YAP1 in BCa tissues with high and low PLAGL2 expression. Representative IHC staining showed that RACGAP1 and nuclear YAP1 expression levels were increased in PLAGL2-overexpressing BCa tissues; conversely, p-YAP1 expression was reduced in PLAGL2-overexpressing BCa tissues (Fig. 8A, B). Notably, BCa patients with high PLAGL2 and high RACGAP1 or with high PLAGL2 and high nuclear YAP1 had a more unfavorable DFS than others (Fig. 8C). Thus, PLALG2 enhances YAP1 activity by upregulating RACGAP1-mediated RhoA GTPase, promotes BCa progression and induces poor prognosis (Fig. 8D).

## Discussion

Recently, mounting evidence has helped elucidate the biological roles of PLAGL2 in various human cancers, including BCa [13], HCC [6], GC [36], breast cancer [37], CRC [7], and gliomas [38]. DJ-1-induced Wnt signaling activation in CRC is dependent on PLAGL2 expression [39]. PLAGL2 functions as a glioma oncogene to impart stem cell-like characteristics in malignant cells [38]. Zhan et al. found the regulatory mechanisms of PLAGL2 in cellular metabolism in glioma [40]. PLAGL2 increases the transcription of ACLY and ELOVL6, leading to enhanced de novo lipogenesis and altered glioma cell behavior. Qu et al. found increased PLALG2 in both BCa tissues and metastatic lymph nodes through IHC staining analysis [13]. In addition, PLAGL2 functions as a crucial regulator of EMT to promote cancer cell invasion and metastasis. PLAGL2 transactivates UCA1, which enhances YTHDF1 by sponging miR-145-5p to regulate Snail1 (a key inducer of EMT) expression and eventually induces GC EMT and metastasis [41]. Similarly, PLAGL2 promotes the migration of GC cells via USP37-mediated deubiquitination of Snail1 [36]. Another EMT activator, ZEB1, is also regulated by PLAGL2 to boost the metastasis of CRC cells in a β-catenin-dependent manner [7]. PLAGL2 can also interact with miRNAs, circRNAs, or lncRNAs to form competing endogenous RNA networks that aggravate malignant phenotypes. For example, PLAGL2 with miR-1270 and circRNA-103809 promotes the development of HCC [42]. Gao et al. also uncovered the PLAGL2/miR-1270/circRNA-SOX4 axis, which is involved in lung adenocarcinoma by activating the WNT signaling pathway [43]. Furthermore, PLAGL2 can promote malignant progression via positive loop regulatory mechanisms, such as the MAPKAPK5-AS1/PLAGL2/HIF-1α and PLAGL2-EGFR-HIF-1/2α loops [6, 44]. However, the biological mechanism of PLAGL2 overexpression in BCa remains unknown and needs further investigation. In our study, we observed that PLAGL2 was upregulated and positively correlated with poor DFS in BCa. Additionally, we explored the biological roles of PLAGL2 and revealed its proproliferative, antiapoptotic, and prometastatic properties in BCa cells.

PLAGL2, acting as a TF and binding directly to the promoter region of downstream targets, was found to regulate the expression of MYCN [45] and Wnt6 [46] by directly binding to their promoters. Additionally, overexpression of PLAGL2 was observed to transactivate the Mpl receptor and collaborate with Cbfβ-SMMHC to trigger leukemia in mice [47]. Our study uncovered a novel transcriptional regulatory mechanism between PLAGL2 and RACGAP1 that is responsible for BCa progression. Overexpressed RACGAP1 restored the proliferation, antiapoptotic effects, and metastasis of BCa cells abrogated by PLAGL2 deficiency, whereas decreased RACGAP1 reversed these abilities upregulated by PLAGL2 overexpression. Our findings were consistent with existing evidence that indicated the procarcinogenic effect of RACGAP1 in many different types of tumor tissues, including GC [48], melanoma [49], and cervical cancer [50]. For example, there is a positive correlation between RACGAP1 expression and clinicopathological features (e.g., older age, tumor size, and advanced stage) of GC [48]. Endothelial barrier function loss and subsequent melanoma transmigration were observed in a study by Zhang et al. [49]. In cervical cancer, RACGAP1 promotes tumorigenicity, migration, and invasion of CC via regulation of the phosphorylation of c-Jun [50]. In a BCa study by Ge et al., a miR-4324-RACGAP1-STAT3-ESR1 feedback loop was observed [51]. These findings collectively suggest that RACGAP1 could be a critical regulator of cancer progression.

Decoding the biological mechanisms of RACGAP1 in regulating cancer progression, researchers have addressed the regulatory axis of RACGAP1-LATS1-YAP1 [24, 33]. In acute kidney injury, the regulatory role of RACGAP1 in YAP1 signaling still exists [52]. Based on this, we hypothesized that PLAGL2 might regulate the Hippo-YAP1 pathway via RACGAP1. Using RNA-seq, immunoblotting, and immunofluorescence, we first found that PLAGL2 regulates the protein levels of p-LATS1 and p-YAP1 and promotes the nuclear translocation of YAP1/TAZ. In addition, increased PLAGL2 increases YAP1/TAZ-TEAD transcriptional activity and the expression profiles of downstream targets. Inhibited PLAGL2 induces the opposite effects. Rescue experiments further support the notion that PLAGL2 promotes BCa by inhibiting the Hippo pathway (specifically, the LATS1 kinase) and increasing YAP1/TAZ activity. Collectively, we proved that PLAGL2 transactivates RACGAP1, which is involved in the regulation of YAP1/TAZ activity. More recently, Li and colleagues reported that RACGAP1-mediated RhoA activation is indispensable to the effects of upstream regulators on downstream YAP1/TAZ activity [24]. Consistently, we discovered that PLALG2 activates YAP1/TAZ activity and promotes BCa progression via RACGAP1-mediated RhoA activation. Hence, a novel molecular mechanism underlying BCa progression was unveiled, and potential therapeutic targets might shed light on effective treatments against BCa.

Cancer cells often have metabolic characteristics distinct from those of normal cells. For example, cell proliferation requires fatty acids for the synthesis of cellular components and secreted molecules [53]. Limiting fatty acid availability can effectively inhibit cancer cell proliferation. Simvastatin is a lipid-lowering agent that impedes the conversion of HMG-CoA to mevalonate by competitively inhibiting HMG-CoA reductase and decreasing cholesterol synthesis. Mechanistically, simvastatin could promote p-YAP by repressing RhoA GTPase activity and inhibit cancer cell proliferation and migration, such as in breast cancer [54] and pancreatic cancer [55]. However, the evidence supporting its treatment for BCa is limited, and the underlying mechanisms remain unclear. In the present study, we showed that simvastatin abrogated the nuclear translocation of YAP1/TAZ, TEAD transcriptional activity, and expression of downstream targets increased by PLAGL2 overexpression, similar to the effects of YAP1 deficiency by siRNA. Our results were consistent with previous findings [56, 57]. Similarly, verteporfin was also found to be sufficient to reverse the proliferative and metastatic phenotype in BCa cells overexpressing PLAGL2.

Development of de novo drugs directly targeting oncogenic TFs (e.g., PLAGL2) is challenging. Alternatively, exploration of drugs targeting the key nodes of the signaling axis may be a promising therapeutic strategy for anticancer therapy. In the present study, simvastatin and verteporfin demonstrated sufficient efficacy in inhibiting BCa progression by targeting key nodes in the PLAGL2/RACGAP1/RhoA GTPase/YAP1 axis. These two inhibitors are not primarily designed for cancer treatment, but their potent anticancer potential has been well documented in various cancers, including prostate cancer [24], head and neck squamous cell carcinoma [58], and breast cancer [59]. Repositioning existing drugs can not only offer a better risk-versus-reward trade-off but also help to increase productivity and lower production costs [60]. Future clinical studies are warranted to investigate the effect of simvastatin or verteporfin in BCa.

In summary, our study showed that upregulated PLAGL2 was linked to unfavorable survival in BCa and that PLAGL2 deficiency significantly inhibited the proliferation and metastasis of BCa cells. Furthermore, PLAGL2 inhibits the Hippo pathway, facilitates the nuclear translocation of YAP1/TAZ, and increases YAP1/TAZ activity via RACGAP1-mediated RhoA activation. Finally, administration of simvastatin or verteporfin had a remarkable inhibitory effect on the progression of BCa. Therefore, PLAGL2/RACGAP1/RhoA GTPase/YAP1 signaling is potentially a crucial target for BCa therapy.

## Supplementary information

Original full length wb blots

Supplementary file

Table S2

## Data Availability

The data supporting the findings of the present study are available from the corresponding author upon reasonable request.
