# Supplementary figures and images for: PLAGL2 promotes bladder cancer progression via RACGAP1/RhoA GTPase/YAP1 signaling
Source: Cell Death Dis. 2023 Jul 15;14(7):433. doi: 10.1038/s41419-023-05970-2 (PMC10349853; doi:10.1038/s41419-023-05970-2)

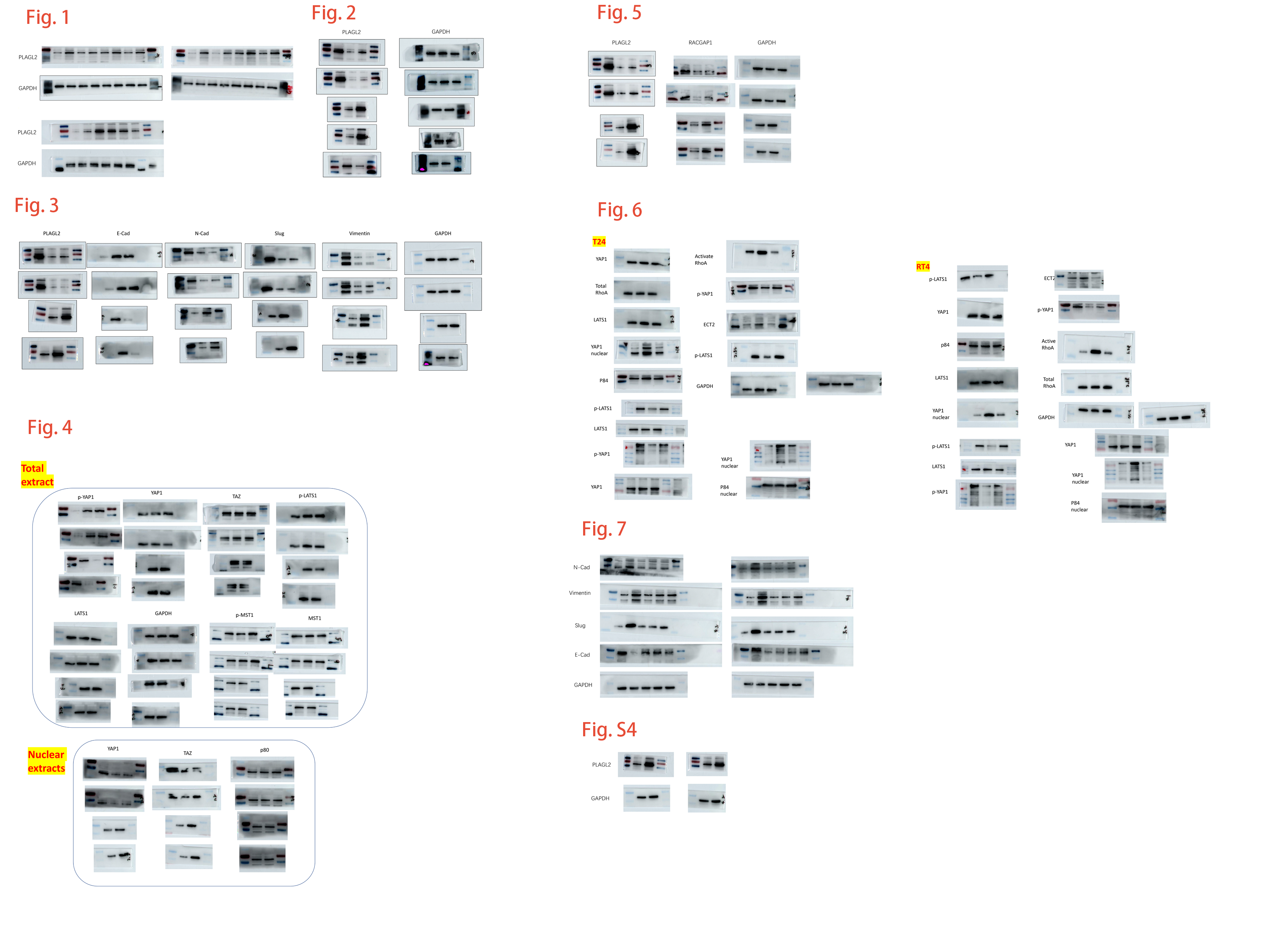

Supplement: Supplementary file 1 — Original full length wb blots [file 41419_2023_5970_MOESM1_ESM.tif]
