# Supplementary material for: PLAGL2 promotes bladder cancer progression via RACGAP1/RhoA GTPase/YAP1 signaling
Source: Cell Death Dis. 2023 Jul 15;14(7):433. doi: 10.1038/s41419-023-05970-2 (PMC10349853; doi:10.1038/s41419-023-05970-2)
Supplement: Supplementary file 2 — Supplementary file [file 41419_2023_5970_MOESM2_ESM.docx]

**
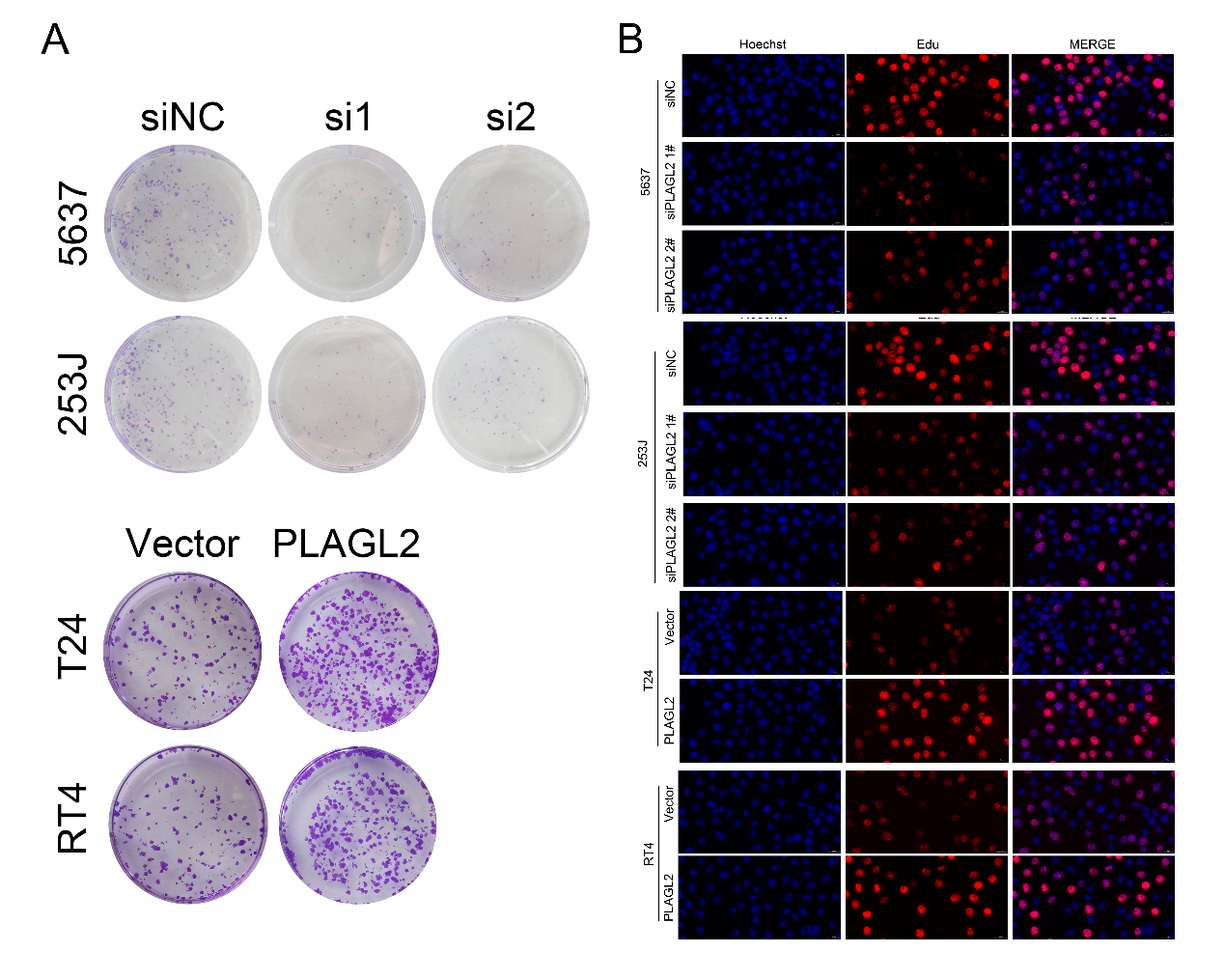
**

**Fig. S1.** PLAGL2 promotes proliferation of BCa *in vitro*. (**A**, **B**) Colony formation (A), and Edu assays (B) knockdown of endogenous PLAGL2 expression in 5637 and 253J cells inhibited, whereas ectopic expression of PLAGL2 in T24 and RT4 cells promoted proliferation.


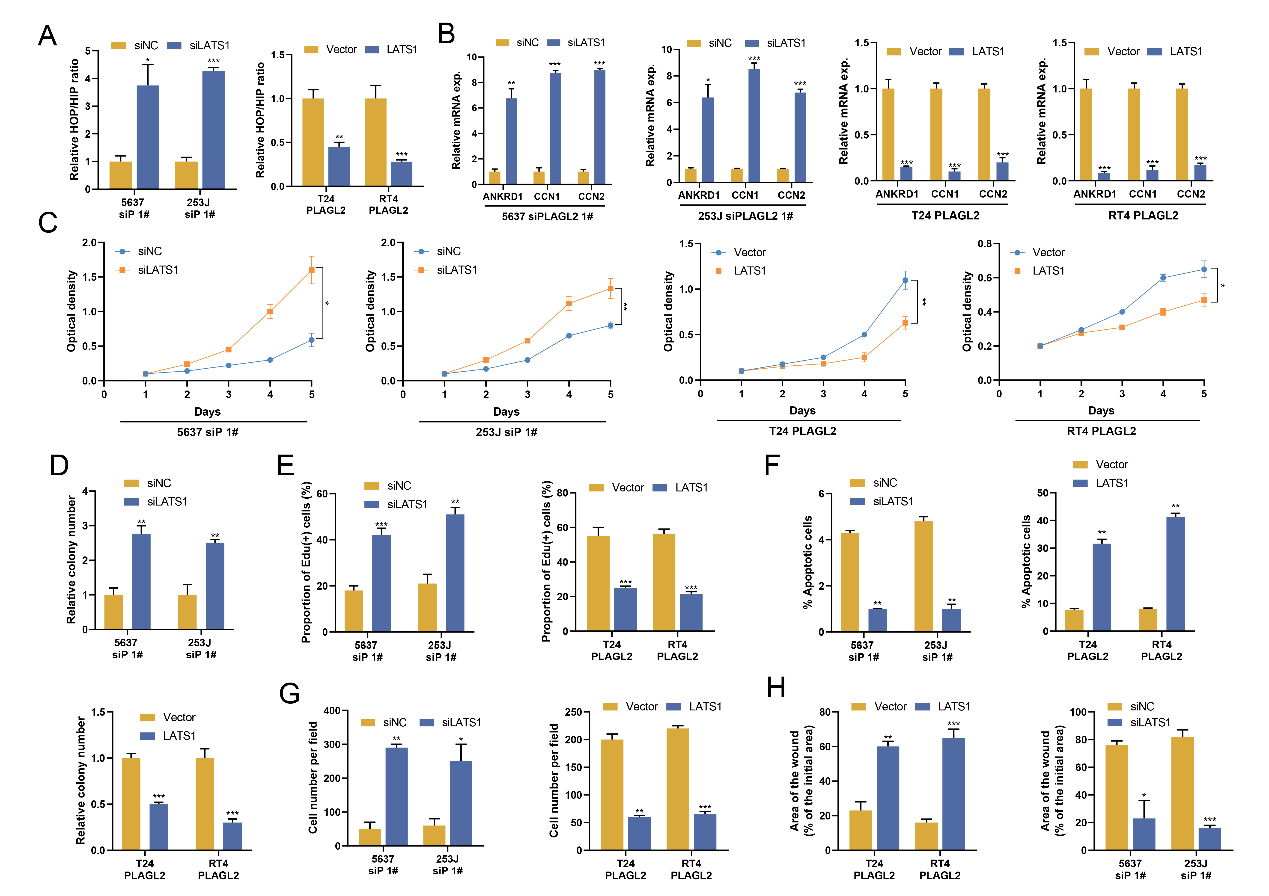


**Fig. S2**. PLAGL2 upregulates YAP1 activity and promotes BCa progression via inhibiting the Hippo pathway. (**A**, **B**) Enhanced LATS1 reversed the YAP1/TAZ-TEAD transcriptional activity (A) and expressions of downstream targets (B) upregulated by PLAGL2 overexpression. But decreased LATS1 restored the activity and expressions inhibited by PLAGL2 deficiency. (**C**-**E**) CCK-8 (C), colony formation (D), and Edu assays (E) showed that enhanced LATS1 inhibited the proliferation increased by PLAGL2 overexpression. But decreased LATS1 restored the ability inhibited by PLAGL2 deficiency. (**F**) Flow cytometry assays showed that enhanced LATS1 promoted the apoptosis inhibited by PLAGL2 overexpression. But deficient LATS1 inhibited the apoptosis increased by PLAGL2 deficiency. (**G**, **H**) The increased metastasis by PLAGL2 overexpression was inhibited by overexpressed LATS1 whereas the decreased metastasis by PLAGL2 deficiency was promoted by inhibited LATS1 as indicated by transwell invasion (G) and wound-healing assays (H). *P < 0.05, **P < 0.01 ***P < 0.001.


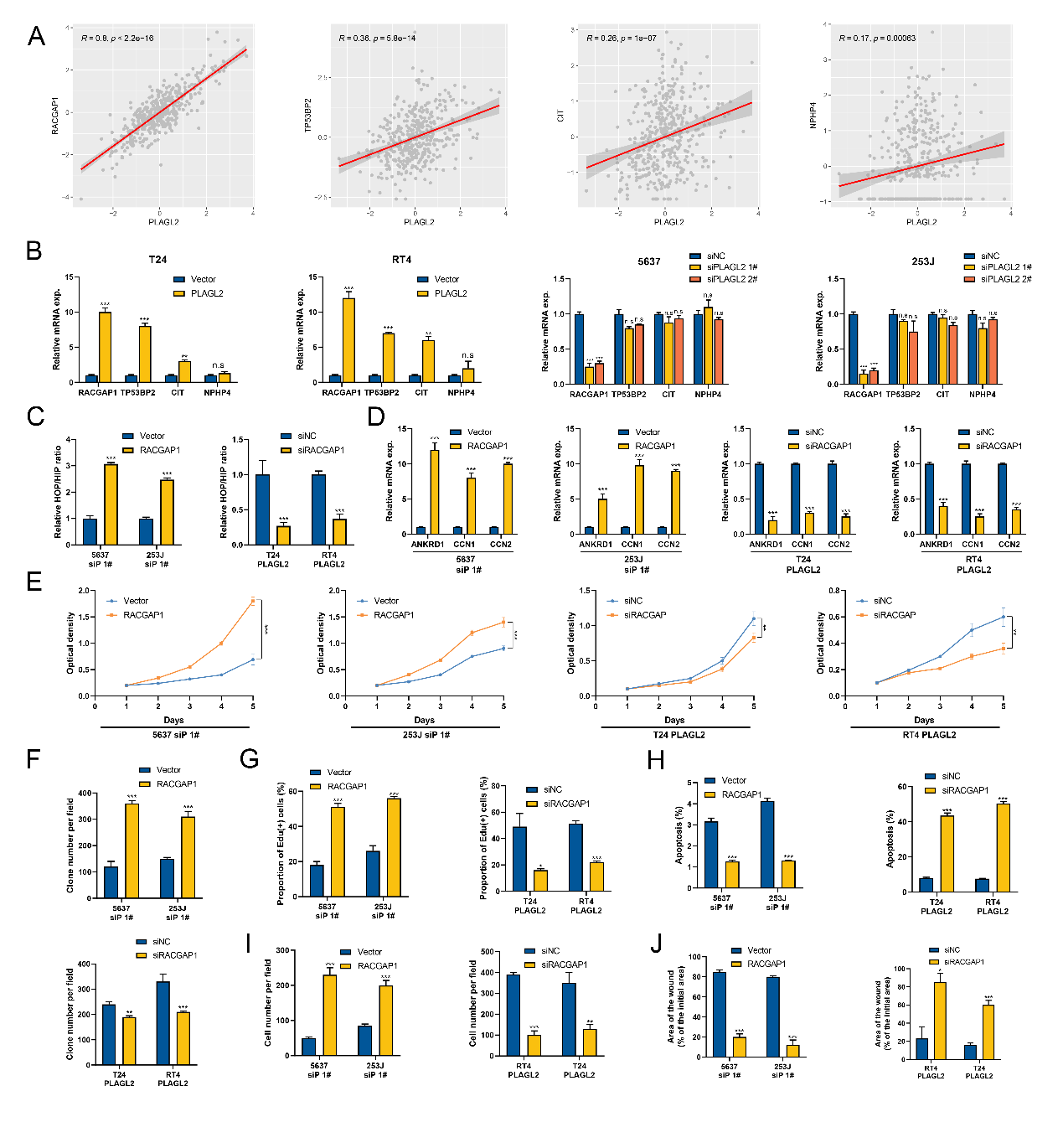


**Fig. S3**. PLAGL2 transcriptionally activates RACGAP1 to repress the Hippo signaling. (**A**) Pearson correlation between PLAGL2 and RACGAP1, TP53BP2, CIT, and NPHP4. (**B**) The effect of PLAGL2 on the mRNA levels of RACGAP1, TP53BP2, CIT, and NPHP4. (**C**, **D**) Enhanced RACGAP1 restored the YAP1/TAZ-TEAD transcriptional activity (C) and expressions of downstream targets (D) downregulated by PLAGL2 deficiency. But decreased RACGAP1 reversed the activity and expressions upregulated by PLAGL2 overexpression. (**E-G**) CCK-8 (E), colony formation (F), and Edu assays (G) showed that enhanced RACGAP1 promoted the proliferation inhibited by PLAGL2 deficiency. But decreased RACGAP1 reversed the ability increased by PLAGL2 overexpression. (H) Flow cytometry assays showed that enhanced RACGAP1 inhibited the apoptosis increased by PLAGL2 deficiency. But deficient LATS1 increased the apoptosis inhibited by PLAGL2 overexpression. (**I**, **J**) The increased metastasis by PLAGL2 overexpression was inhibited by inhibited LATS1 whereas the decreased metastasis by PLAGL2 deficiency was promoted by overexpressed LATS1 as indicated by transwell invasion (I) and wound-healing assays (J). *P < 0.05, **P < 0.01 ***P < 0.001. n.s, no significance.


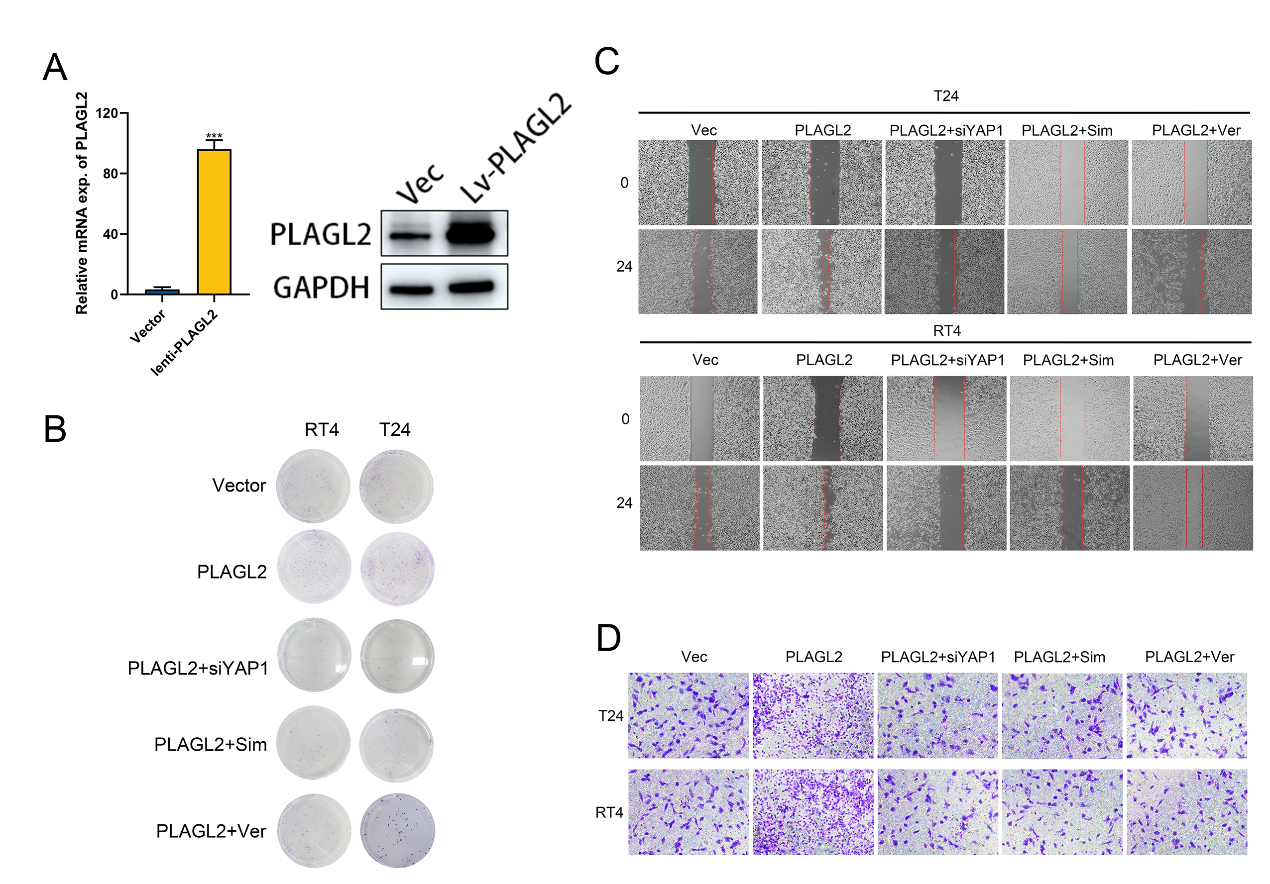


**Fig. S4**. Simvastatin and Verteporfin abrogate the PLAG2-mediated BCa progression. (**A**) The efficiency of lv-PLAGL2 was verified by qRT-PCR and immunoblotting assay. (**B**) Simvastatin and Verteporfin reversed the pro-proliferation of BCa cells induced by PLAGL2 overexpression as indicated by colony formation assays. (**C, D**) Simvastatin and Verteporfin reversed the pro-metastasis of BCa cells induced by PLAGL2 overexpression as indicated by wound-healing (C) and transwell invasion assays (D).

| **Table S1.** List of siRNAs sequences and primers. | |  |
| --- | --- | --- |
| **siRNA** | **Sense (5' - 3')** | **Antisense (5' - 3')** |
| siPLAGL2 1# | GGUUGGUGUGGAUAGCAAAGG | UUUGCUAUCCACACCAACCCA |
| siPLAGL2 2# | CAGUGUAUGUACUGUGAUAAG | UAUCACAGUACAUACACUGGU |
| siYAP1 | GGUCAGAGAUACUUCUUAAAU | UUAAGAAGUAUCUCUGACCAG |
| siLATS1 | GGAGUACUUCAGAAGUUAAUC | UUAACUUCUGAAGUACUCCGA |
| siRACGAP1 | GGUGGAUGUAGAGAUCAAACG | UUUGAUCUCUACAUCCACCUG |
|  |  |  |
| **Primers** | **Forward (5' - 3')** | **Reverse (5' - 3')** |
| **qRT-PCR primers** | | |
| PLAGL2 | GAGTCAAGTGAAGTGCCAATGT | TGAGGGCAGCTATATGGTCTC |
| RACGAP1 | TGCACGTAATCAGGTGGATGT | TGAATCTGTCGTTCCAGCTTTT |
| ANKRD1 | CGTGGAGGAAACCTGGATGTT | GTGCTGAGCAACTTATCTCGG |
| CCN1 | GGTCAAAGTTACCGGGCAGT | GGAGGCATCGAATCCCAGC |
| CCN2 | CAGCATGGACGTTCGTCTG | AACCACGGTTTGGTCCTTGG |
| GAPDH | AGAAGGCTGGGGCTCATTTG | AGGGGCCATCCACAGTCTTC |
| **ChIP-qPCR primers** | | |
| RACGAP1 | GCTCACTAGTCCCTCAGGAAT | GAAGTAGGCAGGAAGTGCTGA |

**Table S2.** Differentially expressed genes (padj < 0.01 & abs(log2FoldChange) > 1) between siPLAGL2 and siNC in 5637 cells.
